# RAFT-Derived Polymethacrylates as a Superior Treatment for Recurrent Vulvovaginal Candidiasis by Targeting Biotic Biofilms and Persister Cells

**DOI:** 10.3389/fmicb.2019.02592

**Published:** 2019-11-07

**Authors:** Xueqing Wu, Sisi Zhang, Xinxin Xu, Laien Shen, Boyun Xu, Wenzhen Qu, Wenyi Zhuang, Katherine Locock, Margaret Deighton, Yue Qu

**Affiliations:** ^1^The Division of Gynecology, Shenzhen University General Hospital, Shenzhen, China; ^2^Department of Obstetrics and Gynecology, Wenzhou Medical University, Wenzhou, China; ^3^CSIRO Manufacturing Flagship, Clayton, VIC, Australia; ^4^School of Chemical and Biomedical Engineering, The University of Melbourne, Melbourne, VIC, Australia; ^5^School of Applied Sciences, RMIT University, Bundoora, VIC, Australia; ^6^Neonatal Intensive Care Unit, The Second Affiliated Hospital and Yuying Children’s Hospital of Wenzhou Medical University, Wenzhou, China; ^7^Department of Microbiology, Monash University, Clayton, VIC, Australia

**Keywords:** RVVC, mouse model, antifungal treatment, RAFT-derived polymethacrylates, biotic biofilms, persister cells, population analysis

## Abstract

**Background:**

Vulvovaginal candidiasis (VVC) is a common infection in need of more effective treatment. Formation of epithelium-associated *Candida* biofilms and the presence of persister cells are among the major contributing factors to the recurrence of this condition. We have previously developed RAFT-derived polymethacrylates that are effective in killing *C. albicans* biofilms *in vitro*. This study aimed to examine the clinical potential of polymethacrylates as antifungals for treatment of recurrent VVC (RVVC).

**Methods:**

A mouse model of VVC was used to establish vaginal epithelium-associated biofilms, using *C. albicans* isolates from VVC/RVVC patients. A comparison was made of the efficacies of polymethacrylates and conventional antifungals, clotrimazole and nystatin, in killing *Candida* in epithelium-associated biofilms *in vivo*. *Ex vivo* biofilms were used for *Candida* population profiling and to quantify persister cells in vaginal epithelia. The potency of polymethacrylates and conventional antifungals against persister cells, either as sole agents or in combination, was assessed.

**Results:**

Polymethacrylates showed negligible local toxicity, resistance to vaginal acidity, and outstanding *in vivo* activity against vaginal epithelium-associated *C. albicans* biofilms. *In vivo* tests polymethacrylates outperformed the conventional antifungals, nystatin and clotrimazole at concentrations 50 times below the over-the-counter concentrations. Using polymethacrylates was associated with fewer persister cells, and better eradication of persister cells pre-selected by conventional antifungals.

**Conclusion:**

This study systematically assessed the clinical potential of RAFT-derived polymethacrylates as an effective treatment for VVC/RVVC in a mouse model. Polymethacrylates effectively killed vaginal epithelium-related *C. albicans in vivo* by specially targeting biotic biofilms and persister cells. Treatment presented negligible local toxicity.

## Introduction

Vulvovaginal candidiasis (VVC) is one of the most common infections in women, affecting up to 75% of those of reproductive age. A 5–8% subset of these women suffer from the very stubborn recurrent VVC (RVVC, at least 3–4 episodes within a 12-month period), which is resistant to currently available treatment (Sobel, 2016). Topical preparations, such as clotrimazole cream or nystatin suppositories are used as first-line antifungals for VVC and RVVC (Wächtler et al., 2011; Choukri et al., 2014). Fluconazole is also recommended by CDC as an effective oral antifungal for induction and maintenance therapy of RVVC. Although most *C. albicans* isolates from RVVC patients remain sensitive to many azoles (Gamarra et al., 2014; Adjapong et al., 2017), suppressive maintenance therapies using azoles often fail to eradicate the pathogen or completely cure the infection (Fan et al., 2015a; Mendling et al., 2015; Grinceviciene et al., 2017), suggesting microbial strategies other than intrinsic resistance might be involved.

Biofilm formation by *C. albicans* on vaginal epithelia has been long recognized as a specific fungal self-protective strategy that leads to antifungal resistance and infection recurrence (Harriott et al., 2010; Muzny and Schwebke, 2015), although its physical presence in the vagina of VVC/RVVC patients was recently questioned (Sobel, 2016; Swidsinski et al., 2019). Persister cells are a small population of “transiently resistant” cells that are often associated with the specific mode of biofilm growth (Lafleur et al., 2006; Lafleur et al., 2010; Yang et al., 2015). It is reasonable to hypothesize that epithelium-associated *C. albicans* biofilms involved in RVVC may harbor persister cells. Treatment of persister cells is known to be troublesome (Lewis, 2010). Conventional antimicrobials, unless used at the very high doses for an extended period, often fail to eradicate persister cells residing in biofilms (Yang et al., 2015).

Fluconazole maintenance suppressive therapy is still a preferred option to treat RVVC caused by fluconazole-sensitive *C. albicans* (Mikamo et al., 2015; Dennerstein, 2017). Other conventional antifungals that have a broad spectrum of activity, such as imidazole and nystatin, have been recommended for RVVC caused by fluconazole-resistant *C. albicans* and other *Candida* species (Fan et al., 2015a, b). Several limitations of using conventional agents to treat RVVC have been reported, including low efficacy in curing RVVC, possible systemic side-effects, and the development of antifungal resistance (Howley et al., 2016; Molgaard-Nielsen et al., 2016). The biofilm growth model of *Candida* species around vaginal epithelia, the possible involvement of persister cells, and the acidic condition of the human vagina, have been shown to reduce the effectiveness of some conventional and newly developed antifungals against *Candida* spp. (Kasper et al., 2015; Muzny and Schwebke, 2015). These factors highlight the importance of developing new, effective and safe antifungal drugs that are able to overcome such hurdles for the treatment of VVC/RVVC.

We have previously developed biocompatible RAFT-derived cationic polymethacrylates and demonstrated their efficacy against *in vitro* biofilms formed by *C. albicans* (Qu et al., 2016). The aim of this study was to systematically evaluate the potential of the polymethacrylates for the treatment of VVC/RVVC, by examining their local toxicity, *in vivo* efficacy in killing biotic biofilms and persister cells, and tolerance to acidic conditions encountered in the human vagina, using a mouse model.

## Materials and Methods

### *C. albicans* Strains, Antifungal Agents and Ethical Approval

Two *C. albicans* clinical isolates and DAY185, a well-known biofilm-producing laboratory strain, were used in this study. The clinical isolates were from patients who visited the first Affiliated Hospital, Wenzhou Medical University with clinically diagnosed uncomplicated VVC and RVVC and were designated as isolates VVC2 and VVC4, respectively. The clinical isolates were identified to a species level using CHROMagar *Candida* medium (CHROMagar, Paris, France) and MALDI Biotyper Identification System (MALDI-TOF MS, BioMérieux, Craponne, France). All strains were stored at −80°C in 15% (v/v) glycerol and streaked onto yeast-peptone-dextrose plates (YPD, 2% peptone, 1% yeast extract, 2% glucose and 80 mg/L uridine) as working stocks. The conventional antifungals for laboratory use, nystatin, clotrimazole, and amphotericin B, were purchased from Merck Pty Ltd. (Nantong, China). The clinically used antifungal preparations, nystatin vaginal suppository and clotrimazole cream were purchased from Polichem S.R.L (Barcelona, Spain) and Bayer Schering Parma AG (Berlin, Germany), respectively. Two polymethacrylates, designated as KL706 and KL708 and with similar compositions to those described previously, were used in this study (Table 1; Qu et al., 2016). The Ethics Review Boards of Wenzhou Medical University approved this study (wydw2016-0214). All animal experiments were carried out in accordance with the National Institutes of Health guide for the care and use of Laboratory animals.

**TABLE 1:** Details of antimicrobial polymethacrylates used in this study, KL-706 and KL-708 (Locock et al., 2014).

**Polymer**	**Structure**	**M*_*n*_* (g/mol)**	**DP**	**MP_methyl_ (%)**
KL-706	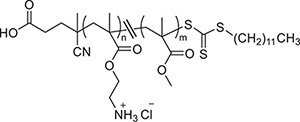	3900	24	29
KL-708	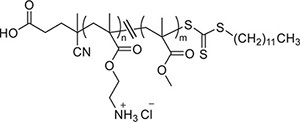	3100	20	48

*M*_*n*_* denotes the number average molecular weight of the polymer, DP the degree of polymerization, or the average monomer units in a polymer chain, and MP_*methyl*_, the mole percentage of methyl side chains in the molecule, in this case compared with amino side chains.*

### Formulation of Antimicrobial Polymethacrylate Hydrogels

Both polymethacrylates were formulated to final concentrations of 20, 40, and 80 mg/L, respectively in hydrogel preparations using a proprietary Carbopol^®^ 940 polymer as follows: Synperonic^TM^ PE/F 127 emulsifier (10 g) was dissolved in water (429 mL) with heating and then cooled to room temperature before the addition of a stock solution of polymethacrylate (0.01–0.04 g) in ethanol (5 mL) and wetted out completely. To do this, the solution was stirred vigorously before the addition of propylene glycol (50.0 g), followed by the slow addition of Carbopol^®^ 940 polymer (5.00 g). The final mixture was brought to pH 7.0 using triethanolamine (0.75 g).

### Qualitative Determination of the Intravaginal Dynamics of Polymethacrylates

Release of polymethacrylates from the formulated hydrogel in the mouse vagina over a time course was determined, using a previously published method (Zhang et al., 2017). Hydrogel loaded with rhodamine-labeled KL708 at 80 mg/L was administered to mice via an intravaginal pathway. Animals were sacrificed at 1, 2, 8, 12, 24, and 48 h and the entire vagina was collected and washed with saline. Fluorescent images of the vagina were taken using an IVIS spectrum *in vivo* imaging system at 550 nm.

### *In vivo* Cervicovaginal Toxicity and Inflammation Assessment

A mouse model was used to assess cervicovaginal toxicity and local inflammation after polymethacrylate exposure (Catalone et al., 2004). BABL/C female mice of 6–8 weeks old were hormonally synchronized 3 days prior to the experiment with a subcutaneous injection of 0.1 mg of estrogen (17β-estradiol; Sigma) dissolved in 0.1 mL sesame oil. Anesthetized mice received an intravaginal treatment (100 μL) of either unformulated hydrogels (negative control) or KL708-hydrogel. PBS was used as the second negative control to document the normal tissue architecture and inflammation status in the cervicovaginal mucosa. Mouse vaginal lavage fluids were collected and the vaginal tissue was surgically excised after sacrificing the animals at 48 h post-treatment. Tissues were formalin-fixed and embedded in paraffin using standard procedures. Gross morphological analyses of the cervicovaginal mucosa were performed on tissues stained with hematoxylin and eosin (H&E) using an Olympus IX81 microscope. Concentrations of two representative inflammatory effectors in vaginal lavage fluids, the innate cytokine IL-1β (Netea et al., 2010) and the alarmin S100A8 (Vogl et al., 2014), were analyzed using commercially available enzyme-linked immunosorbent assay (ELISA) kits per manufacturers’ instructions. All samples were measured in duplicate, and the assay was repeated three times.

### *In vivo* VVC Model and Cultivation of Mouse Vaginal Epithelium-Associated *Candida* Biofilms

Vulvovaginal candidiasis was induced in mice essentially as described by Harriott et al. (2010). *C. albicans* cells were grown for 20 h in YPD (200 rpm, 30°C) and resuspended to a density of ∼ 7 × 10^6^ CFU/mL. One-hundred microliters of *Candida* suspension was used to infect mice via the intravaginal pathway. Infected vaginal tissue was surgically collected after euthanizing animals 3 days post-infection. *C. albicans* biofilms that formed on vaginal epithelia were qualitatively examined using scanning electronic microscopy (SEM).

### Antifungal Susceptibility Tests for Planktonic Cells and Cells Residing in Epithelium-Associated Biofilms

Broth dilution susceptibility testing was carried out for *C. albicans* cells grown as planktonic cultures, by following the CLSI guideline M27-A3, except RPMI-1640 at pH 4.0 and pH 7.2 were used respectively, as growth media. Polymethacrylate solutions at concentrations ranging from 1 to 128 mg/L were tested. For *in vivo* biofilm antifungal susceptibility testing, *C. albicans* epithelium-associated biofilms were established in the mouse vagina as described above. Nystatin vaginal suppository and clotrimazole cream at 4000 mg/L (OTC preparations), and hydrogels loaded with KL706 and KL708 respectively, at 20, 40, and 80 mg/L were delivered as a single dose to infected vagina via an intravaginal pathway. Treatment lasted for 24 h before infected animals were sacrificed, and the entire vagina was surgically excised. The vaginal tissues were weighted with a Mettler Toledo PB603-S milligram balance, washed three times with PBS, and homogenized with a tissue homogenizer. Viable counts were performed for tissue suspensions by plating an aliquot onto YPD plates followed by further incubation at 35°C for 48 h. Colony-forming-unit (CFU) of *Candida* cells per gram of vaginal tissue (CFU/g) was calculated for quantitative assessment of fungal biofilm survivors after antifungal treatment. Mouse vagina treated with PBS and blank hydrogels served as negative controls. Six biological repeats were carried out for this experiment.

### Population Profiling of Infected Vaginal Tissues and *ex vivo* Quantification of Persister Cells

Profiling the population of *C. albicans* cells within infected tissues was carried out using a previously published method, with modification (Yang et al., 2015). Approximately 0.3 g of infected vaginal tissues were homogenized with a tissue homogenizer in 1 mL of RPMI-1640. Suspensions of *Candida* cells were adjusted to a density of ∼ 1 × 10^8^ CFU/mL with RPMI-1640 and a hemocytometer, and further confirmed with CFU-based viable counts. One-hundred microliters of fungal suspension was challenged with nystatin, clotrimazole, and KL706 prepared in RPMI-1640 at increasing concentrations ranging from planktonic MIC to 1024 mg/L for clotrimazole, 2048 mg/L for nystatin, and 256 mg/L for KL706. Our preliminary study found such high concentrations were effective against vaginal epithelium-associated *C. albicans* biofilms *ex vivo*. Viable counts were performed after exposing fungal suspensions to antifungal agents at various concentrations for 24 h, and the highest concentrations for an extra 24 h (48 h in total) and 48 h (72 h in total). To avoid antifungal carryover, the antifungal treated suspensions were centrifuged at 3000 *g* for 5 min, washed with PBS, and resuspended to the same volume of YPD broth. Viable counts were performed by plating serially diluted aliquots onto YPD plates followed by incubation at 35°C for 72 h to maximize the recovery of persister cells. The lowest concentration of antifungals and the shortest treatment duration that led to the lowest number of fungal survivors were chosen as the antifungal regimens for persister cell quantification. The percentage of persister cells were calculated as follows: (fungal density after antifungal treatment)/(fungal density before antifungal treatment) × (100%).

### Potency of Polymethacrylates in Further Killing Persister Cells Pre-selected by Conventional Antifungals

As persister cells residing in biofilms might be antimicrobial-dependent (Yang et al., 2015), we further evaluated the effectiveness of polymethacrylates in killing persister cells that have been pre-selected by conventional antifungals. A population of ∼1 × 10^8^ CFU/mL of fungal cells from infected vaginal tissues were treated with amphotericin B at 100 mg/L for 24 h, washed with PBS to remove the amphotericin B, and then subjected to treatment using nystatin (2048 mg/L), or clotrimazole (1024 mg/L), or KL706 (80 mg/L) or KL708 (80 mg/L) for another 24 h. Viable counts were carried out to recover and quantify survivor persister cells as described earlier. To increase the detection sensitivity, a one mL volume of the treated suspension was pelleted, washed three times with PBS, and resuspended into 100 μL of PBS, followed by spreading and recovering on YPD plates. The experiment was repeated three times in duplicate.

### Data Analysis and Statistical Methods

One-way ANOVA or a non-parametric test (Mann-Whitney *U* test) was carried out to compare two means, depending upon the data distribution. Statistical significance was assumed at the *p*-value of less than 0.05. Data analysis was performed using Minitab 16 (Minitab, State College PA, United States).

## Results

### Formulation of Polymer Loaded Hydrogels and Intravaginal Sustainability of RAFT-Derived Polymethacrylates

**FIGURE 1:**
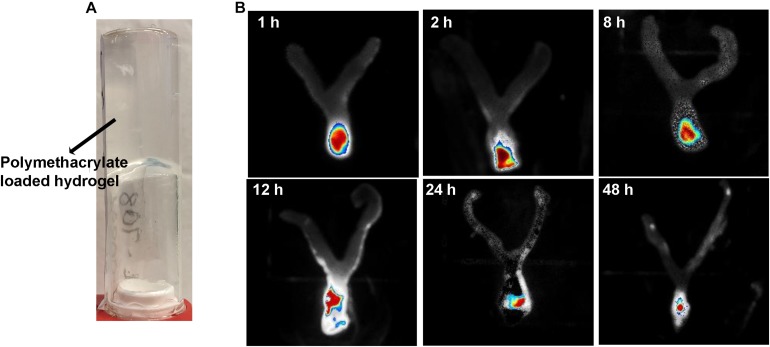
Intravaginal retention and slow decay of polymethacrylates formulated as hydrogel *in vivo*. Fluorescence-labeled polymethacrylate KL-708 was formulated into hydrogels **(A)** and administered intravaginally to mice. Retention of guanylated polymer in mouse vagina was monitored *in situ*, using an *in vivo* IVIS spectrum imaging system at different time points of 0, 2, 8, 12, 24, and 48 h **(B)**. Substantial amounts of polymethacrylates were found in mouse vagina even at 48 h post-administration.

Figure 1A is an example of a formulated hydrogel of polymethacrylate KL708. Figure 1B showed a gradual diminution of rhodamine-labeled KL708 from the hydrogel for a 48-h period after infusion into the mouse vagina. The slow decay of fluorescence intensity in the vagina supported the stability, for at least 48 h, of such a drug delivery system in the vaginal environment and a prolonged contact between polymethacrylate and the vaginal epithelia, paving the foundation for intravaginal application of this system.

### Minimum Cervicovaginal Toxicity of RAFT-Derived Polymethacrylates

**FIGURE 2:**
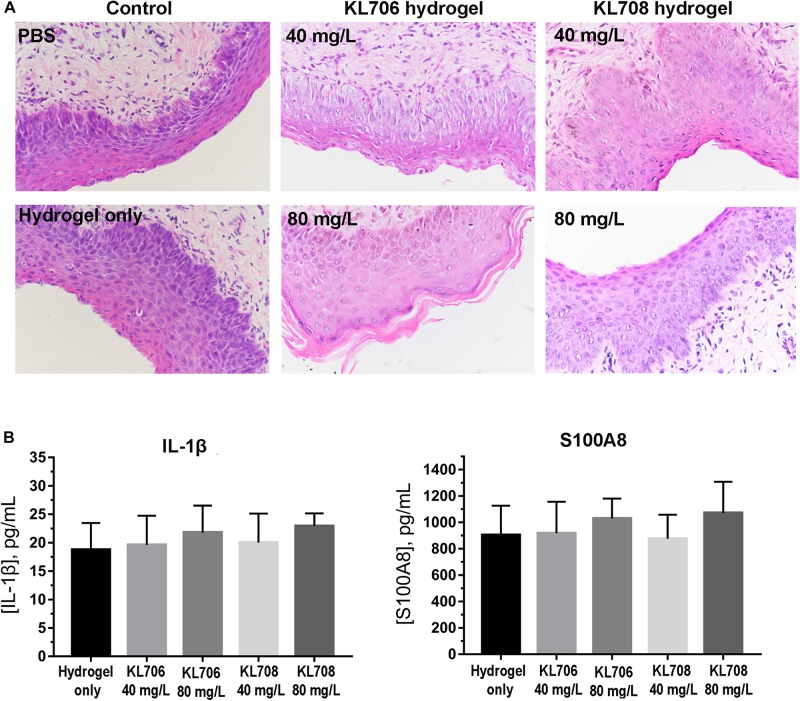
Cervicovaginal toxicity of polymethacrylates and local inflammation. **(A)** Histopathological presentation of mouse vaginal tissues after exposure to polymethacrylate at 40 and 80 mg/L for 48 h. HE staining showed neglectable morphological changes of vaginal epithelia and underlying tissues. **(B)** Inflammation responses induced by polymethacrylate-loaded hydrogels. Inflammatory cytokine IL-1β and alarmin S100A8 were used as indicators.

Toxicity of polymethacrylates to cervicovaginal tissues was determined by examining the morphology of vaginal epithelia and local inflammation. Hematoxylin and eosin staining showed no significant change in the gross appearance of the vaginal mucosa after exposure to hydrogels loaded with KL708 at 40 mg/L or 80 mg/L, or KL706 at 40 mg/L for 48 h, when compared with hydrogel or non-hydrogel PBS controls (Figure 2A). No apparent epithelial disruption in the mouse vagina was noticed when challenged with these products. Vaginal epithelia remained protected by a covering of keratin, and the integrity of the squamous epithelia appeared intact (Figure 2A). Minor sloughing was observed when KL-706 was used at 80 mg/L, however this involved only the upper epithelia (Figure 2A). No significant inflammatory responses were induced in mouse vagina by polymethacrylate-loaded hydrogels, even at a high polymer concentration of 80 mg/L, as indicated by the level of inflammatory cytokine IL-1β and alarmin S100A8 (Figure 2B).

### Acidic Vaginal Environment Did Not Compromise Anti-candida Activity of Polymethacrylates

Minimum inhibitory concentrations were determined for both conventional antifungals and polymethacrylates, using the standard broth-dilution method but with RPMI prepared at a neutral pH (7.2) and a pH comparable to that in the human vagina (4.0) respectively. All but one of the MICs derived for the polymethacrylates were identical under acidic conditions, indicating a high tolerance of these polymers to acidic conditions found within the human vagina (Table 2). The acidic condition, however, significantly compromised antifungal activities of conventional drugs, by increasing their MICs by 4–16-fold (Table 2).

**TABLE 2:** *In vitro* susceptibility of *C. albicans* strains to conventional antifungals and polymethacrylates at different pHs.

**Antifungals**	**MIC (mg/L) for *C. albicans* isolates**					
	
	**DAY185**	**Fold change**	**VVC2**	**Fold change**	**VVC4**	**Fold change**
**pH = 7.2**						
Nystatin	4		2		4	
Clotrimazole	0.5		2		1	
KL706	16		16		16	
KL708	16		16		32	
**pH = 4.0**						
Nystatin	16	4	32	16	32	8
Clotrimazole	8	14	8	4	16	16
KL706	32	2	32	2	32	2
KL708	32	2	32	2	32	1

### Polymethacrylates Were Superior to Conventional Antifungals in Killing Epithelium-Associated Biofilms in the Vagina

**FIGURE 3:**
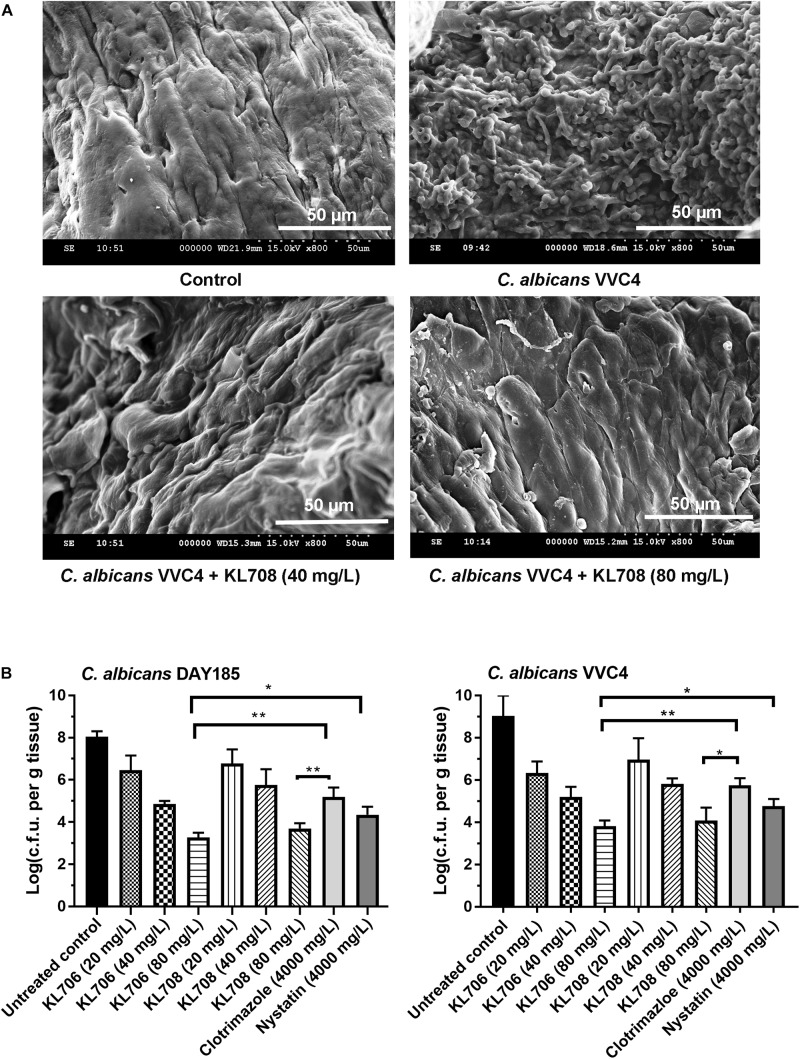
Antifungal activities of polymethacrylates against *C. albicans* grown as vaginal epithelium-associated biofilms. **(A)** Scanning electron microscopy of epithelium-associated biofilms formed by *C. albicans* VVC4 in the mouse vagina, before and after treatment with KL708 at 40 and 80 mg/L. **(B)** Quantitative assessment of anti-infective efficacy of polymethacrylates against vaginal epithelium-associated *C. albicans* biofilms. *C. albicans* cells grown as biotic biofilms on mouse vaginal epithelia were treated with polymethacrylates at 40 and 80 mg/L and conventional antifungal drugs at clinical doses. Mouse vaginal tissue was removed after treatment and homogenized for viable counts. Survivors were recovered after 72 h incubation on YPD plates. Polymethacrylates at non-toxic concentrations (80 μg/mL) demonstrated a significantly higher activities in killing epithelium-associated biotic *C. albicans* biofilms than conventional antifungals. No difference was found between the two negative controls (PBS control and hydrogel-only control) and only that of hydrogel-only was shown. ^∗^*p* < 0.05, ^∗∗^*p* < 0.01.

We first examined *in vivo* biofilm formation of *C. albicans* on mouse vaginal epithelia at 48 h post-infection. A characteristic epithelium-associated biofilm structure was observed with SEM, consisting of yeast and hyphal cells embedded in vaginal mucosae (Figure 3A, with the clinical isolate VVC4 as an example). We further examined the antifungal efficacy of all agents against such epithelium-associated biofilms formed by VVC4 and laboratory strain DAY185. KL706 and KL708 at 40 mg/L were effective in killing epithelium-associated biofilms formed by either *C. albicans* DAY185 or VVC4 (Figures 3A,B). KL706 at 80 mg/L, a concentration 50 times less compared to that of conventional antifungals tested, killed significantly more biofilms than either nystatin or clotrimazole at 4000 mg/L (*p* < 0.05 and *p* < 0.01, respectively). KL708 at 80 mg/L also showed a higher potency than clotrimazole at 4000 mg/L in killing epithelium-associated *C. albicans* biofilms in the mouse vagina (Figure 3B, *p* < 0.05).

### Polymethacrylate Treatment of Epithelium-Associated *C. albicans* Biofilms Resulted in Fewer Persister Cells Than Conventional Antifungals

**FIGURE 4:**
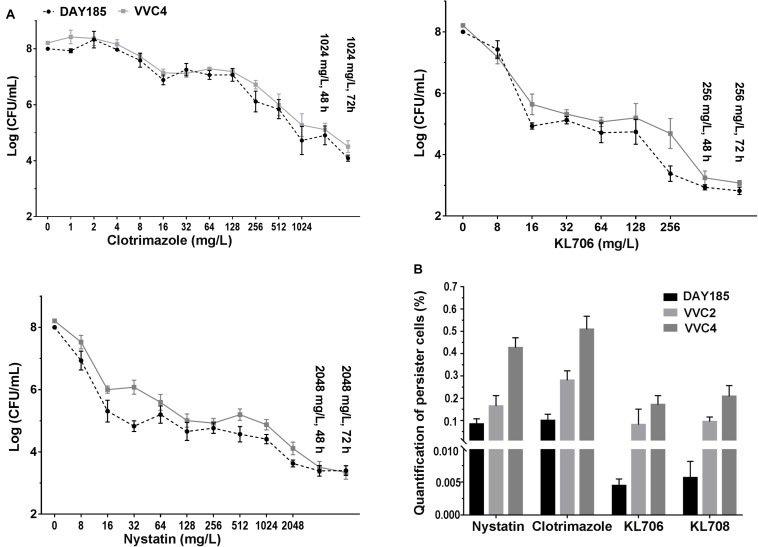
**(A)** *Ex vivo* population profiling of vagina epithelium-associated *C. albicans* biofilms using different antifungal agents. Mice were infected with *C. albicans* via the intravaginal pathway. After 3 days, infected vaginal tissues were harvested and homogenized. Fungal suspensions containing persister cells were challenged with antifungals at increasing concentrations for 24, 48, and 72 h. Experimental results identified a small population remaining tolerant to antifungals at very high concentration, even for an extended treatment period (48 or 72 h). Polymers KL706 only required a relatively low concentration of 256 mg/L and 24–48 h to reach the persister cell plateau, however the conventional antifungal agents need a higher concentration of 1024 mg/L or 2048 mg/L to reach this plateau. **(B)** Proportions of *C. albicans* persister cells in epithelium-associated biofilms selected by different antifungal agents. Total persister cells were isolated by exposing homogenized mouse vaginal tissues infected with *C. albicans* to antifungal agents at a specific concentration (1024 mg/L for clotrimazole, 2048 mg/L for nystatin, and 256 mg/L for KL706 and KL708, based on population analysis data). Such regimens have been found to be able to isolate persister cells from infected vaginal tissues. The proportion of persister cells was calculated as the proportion of survivor cells out of total population. Shown are the average of three to four biological repeats in duplicate.

Population analysis profiling was performed to determine antifungal regimens (concentration and duration) that can be used to successfully isolate and quantify persister cells residing in epithelium-associated *C. albicans* biofilms. Typical three-subpopulation patterns were established when nystatin and KL706 were used as the selecting agents (Figure 4A): Over 99.9% of biofilm cells were susceptible and responded to both agents at 16 mg/L; a small population of cells demonstrated tolerance to nystatin and KL706 at higher concentrations (16–1024 mg/L for nystatin, and 16 128 mg/L for KL706) but could be killed if antifungal concentration and/or exposure time were further increased; a very small population of cells survived the most potent antifungal regimens, such as nystatin at 2048 mg/L and KL706 at 256 mg/L for 48 or 72 h. In contrast; a gradually descending pattern was found when fungistatic clotrimazole was used to select biofilm persister cells (Figure 4A). Thus, the regimens chosen to isolate persister cells in epithelium-based biofilms grown in the mouse vagina were 2048 mg/L × 48 h for nystatin, 256 mg/L × 48 h for KL706 and KL708, and 1024 mg/L × 72 h for clotrimazole.

In general, fewer persister cells were detected after exposing vaginal epithelium-associated biofilm cells to KL706 or KL708 than to clotrimazole or nystatin (Figure 4B and Supplementary Table S1). When epithelium-associated biofilms formed by *C. albicans* VVC4 and VVC2 were studied, average proportions of persister cell selected by KL706 and KL708 reached 0.08 ± 0.03% (mean ± SEM) and 0.10 ± 0.01%, and 0.17 ± 0.02% and 0.21 ± 0.02%, respectively. Conventional antifungal drugs nystatin isolated 0.16 ± 0.02% and 0.28 ± 0.02% persister cells, and clotrimazole selected 0.43 ± 0.02% and 0.51 ± 0.02% from biofilms formed by VVC4 and VVC2 respectively (Figure 4B). The same trend was noticed when *C. albicans* laboratory reference strain DAY185 was studied. It was also noticed that vaginal epithelium-based biofilms formed by clinical isolates VVC4 and VVC2 harbored more persister cells than *C. albicans* DAY185, a clinically relevant isolate related to systemic infections, regardless of selecting antifungals used.

### Polymethacrylates Demonstrate Activity Against Persister Cells Following Conventional Antifungal Treatment

**FIGURE 5:**
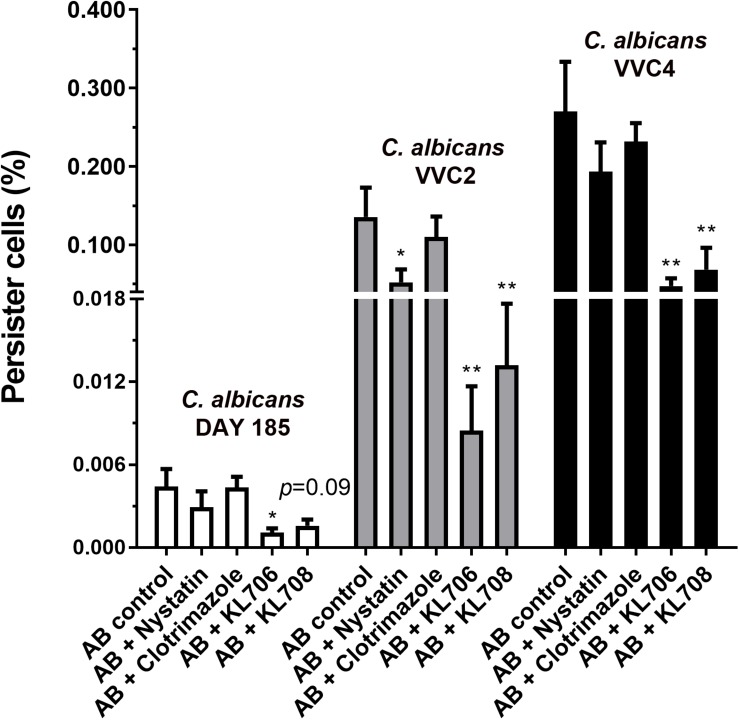
Polymethacrylates demonstrate activity against persister cells pre-selected by conventional antifungals. *Ex vivo* biofilms were used to examine the effectiveness of polymethacrylates in killing persister cells pre-selected by conventional antifungal agent amphotericin B. Mice were infected with *C. albicans* via the intravaginal pathway. After 3 days, infected vaginal tissues were harvested and homogenized. Fungal suspensions containing persister cells were challenged with amphotericin B at 100 mg/L for 24 h, followed by polymethacrylates (128 mg/L), nystatin (1024 mg/L) or clotrimazole (1024 mg/L) for another 24 h. Viable counts were carried out to determine the number of survivor fungal cells. Polymethacrylates at 128 mg/L showed a significantly higher fungicidal activity relative to the conventional agents such as nystatin and clotrimazole at 1024 mg/L. AB: amphotericin B; AB + nystatin: fungal suspension was pre-treated with amphotericin B (100 mg/L) for 24 h and then by nystatin at 1024 mg/L for another 24 h. ^∗^*p* < 0.05, ^∗∗^*p* < 0.01.

*Ex vivo* biofilms were used to examine the effectiveness of polymethacrylates in killing persister cells pre-selected by conventional antifungal agent amphotericin B. When challenging persister cells preselected with amphotericin B at 100 mg/L, polymethacrylates at 128 mg/L showed a significantly higher fungicidal activity relative to the conventional agents, nystatin and clotrimazole at 1024 mg/L (Figure 5). KL706 further killed 75, 94, and 82% of pre-selected persister cells of DAY185, VVC2, and VVC4, and KL708 eradicated 64, 90, and 75% of these persister cells. This opens the door for novel and more efficient combination therapies for RVVC, using conventional antifungals sequentially combined with polymethacrylates.

## Discussion

The current antifungal regimens for VVC/RVVC, recommended by CDC and based on 2015 Sexually Transmitted Diseases (STD) Treatment Guidelines, have been widely followed by general practitioners and gynecologists. Among the conventional antifungals recommended by CDC, imidazoles (including clotrimazole, miconazole and econazole), the polyene nystatin, and fluconazole have been on the market for more than 35 years and still remain effective against acute VVC. Their effectiveness for RVVC, however, are suboptimal, as recurrence of infection is common after an initial course of fluconazole or clotrimazole and ongoing antifungal maintenance therapy is often required (Tapia et al., 2017; Crouss et al., 2018). This is possibly due to the low efficacy of conventional antifungals in eradicating biotic biofilms and persister cells that have grown on vaginal epithelia and in underlying tissues.

We have recently developed a new class of antimicrobial polymethacrylates that have shown high potency against *in vitro C. albicans* biofilms (Qu et al., 2016). This study was designed to examine clinical potential of polymethacrylates as an effective treatment for VVC/RVVC. In the current study, polymethacrylates demonstrated several properties as topical agents for VVC/RVVC, including high efficacy in killing epithelia-based *C. albicans* biofilms and persister cells, ease of formulation as a hydrogel for topical application, and low toxicity to local vaginal tissue. Unlike other newly proposed antifungal substances from natural sources that often need to be combined with conventional agents such as fluconazole or miconazole for high *in vivo* effectiveness (Gao et al., 2016; Esposito et al., 2018; Fernandes Costa et al., 2019), polymethacrylates provide a distinct advantage as they can be used as a sole therapy. They also outperformed clotrimazole and nystatin, two market leading antifungals, at 50 times less the equivalent clinical concentration in killing biotic *C. albicans* biofilms grown on vaginal epithelia. Moreover, polymethacrylates were able to maintain their activity in the acidic human vaginal environment, a major hurdles for drug development, since antifungals are often less effective under acid conditions (Ilkit and Guzel, 2011). Similar to valproic acid (Chaillot et al., 2017), both KL706 and KL708 demonstrated pH-independent antifungal efficacy, exhibiting only a 2-fold shift at pH 4.0 compared with pH 7.2, superior to many conventional antifungals and even the newly developed antifungal CD101 (Boikov et al., 2017).

The *in vivo* efficacy of polymethacrylates for VVC/RVVC found in this study is thought to be based on their unique activity against biotic biofilms and persister cells formed by *C. albicans*. Antifungal resistance stemming from the formation of *Candida* biofilms in VVC has been noted by several studies, mostly based on antifungal susceptibility tests using 96-well microplate biofilm assays (Gao et al., 2016; Sherry et al., 2017). Abiotic biofilms developed in 96-well microplates, however, might differ from biotic epithelium-based biofilms in many aspects, including their resistance to antimicrobial agents (Bjarnsholt et al., 2013). We carried out antifungal susceptibility testing on *Candida* biofilms established in a mammalian vagina that closely mimic the clinical environment of human VVC. Though the mouse vagina differs from that of women in various physical aspects and may have different responses to *Candida* infections, it is still the preferred *in vivo* model for drug development, in the context of cost-effectiveness and labor-intensity (Vecchiarelli et al., 2015).

Harriott et al. (2010) successfully demonstrated vaginal epithelium-based *Candida* biofilms using a mouse VVC model and raised the hypothesis that biofilm formation by *Candida* might be an initiating event for VVC (Harriott et al., 2010). The role of biofilms in the pathogenesis of VVC/RVVC was recently challenged by Swidsinski et al. (2019). Swidsinski et al. (2019) used fluorescent *in situ* hybridization (FISH) on vaginal biopsies from VVC patients and found no evidence of typical biofilm structures on vaginal epithelia or underlying tissues. It should be noted that biotic biofilms found *in vivo* often fail to present the typical three-dimensional mushroom structure, a biofilm morphology frequently reported by *in vitro* studies. *In vivo* biofilms uniquely grow as monolayers or microcolonies (Bjarnsholt et al., 2013), and detections of such structures often require experimental methods of higher resolutions such as SEM. From microbiological and clinical aspects, it is also reasonable to assume the involvement of *Candida* biofilms and persister cells in VVC/RVVC, as the recurrence pattern of RVVC coincides with the model of recurrent biofilm infections and persister cells proposed by Lewis (2010). Clinical isolates of *C. albicans* from the same RVVC patients over different periods of time, despite therapy, were found to be genetically identical, supporting the possible involvement of persister cells in the recurrence of RVVC (Fodor et al., 2002).

One of the major limitations of this study is that no other *Candida* species were included. Non-*C. albicans Candida* species have recently emerged as important etiological agents of VVC/RVVC (Richter et al., 2005; Gamarra et al., 2014). In contrast to *C. albicans*, some non-*C. albicans Candida* species are unable to form typical biofilms *in vitro* or *in vivo*, or are intrinsically resistant to many conventional antifungals (Guzel et al., 2013). A future study is required to examine the effect of polymers against VVC/RVVC caused by other *Candida* species.

## Conclusion

This study systematically assessed the clinical potential of RAFT-derived polymethacrylates as an effective treatment for VVC/RVVC in a mouse model. Polymethacrylates effectively killed vaginal epithelium-related *C. albicans in vivo* by specially targeting biotic biofilms and persister cells, outperforming other market leading antifungals. Treatment presented negligible local toxicity.

## Data Availability Statement

The datasets generated for this study are available on request to the corresponding author.

## Ethics Statement

The Ethics Review Boards of Wenzhou Medical University approved this study (wydw2016-0214). All animal experiments were carried out in accordance with the National Institutes of Health guide for the care and use of Laboratory animals.

## Author Contributions

YQ and XW conceived and designed the study. SZ, XX, LS, BX, WQ, WZ, KL, and YQ carried out the experiments. YQ, SZ, XW, and MD performed the data analysis. YQ wrote the manuscript. YQ, MD, and XW edited the manuscript. All authors reviewed the manuscript and provided critical comments.

## Conflict of Interest

The authors declare that the research was conducted in the absence of any commercial or financial relationships that could be construed as a potential conflict of interest.
