# Supplementary material for: RAFT-Derived Polymethacrylates as a Superior Treatment for Recurrent Vulvovaginal Candidiasis by Targeting Biotic Biofilms and Persister Cells
Source: Front Microbiol. 2019 Nov 7;10:2592. doi: 10.3389/fmicb.2019.02592 (PMC6853869; doi:10.3389/fmicb.2019.02592)
Supplement: Supplementary file 1 [file Table_1.DOCX]

**Supplementary table 1: Statistical comparisons between persister cells in epithelium-associated biofilms selected by different antifungals.**

|  |  | ***P-*value** |  |
| --- | --- | --- | --- |
|  | Nystatin | Clotrimazole | KL706 |
| ***C. albicans* DAY185** |  | | |
| Clotrimazole vs | 0.361 | / | / |
| KL706 vs | <0.001* | <0.001 | / |
| KL708 vs | <0.001 | <0.001 | 0.348 |
| ***C. albicans* VVC2** |  |  |  |
| Clotrimazole vs | 0.003 | / | / |
| KL706 vs | 0.058 | 0.001 | / |
| KL708 vs | 0.017 | <0.001 | 0.061 |
| ***C. albicans* VVC4** |  |  |  |
| Clotrimazole vs | 0.037 | / | / |
| KL706 vs | <0.001 | <0.001 | / |
| KL708 vs | <0.001 | <0.001 | 0.023 |

* is the *P-*value for statistical significance when persister cells were selected by KL706 and nystatin respectively from mouse vaginal epithelium-associated biofilms and quantitatively compared.
